# Causal Associations of Obstructive Sleep Apnea With Cancer Risk: A Mendelian Randomization Study

**DOI:** 10.1002/brb3.70462

**Published:** 2025-05-05

**Authors:** Xiaoyu He, Xiaoting Ye, Kaiqian Yang, Zhenjiang Li

**Affiliations:** ^1^ Department of Hematology The Second Affiliated Hospital of Nanchang University Nanchang China; ^2^ People's Hospital of Zhenhai Ningbo Zhejiang China; ^3^ Department of Hematology The First Affiliated Hospital of Wenzhou Medical University Wenzhou China

**Keywords:** cancer, causal association, Mendelian randomization, obstructive sleep apnea

## Abstract

**Background:**

Observational studies have associated obstructive sleep apnea (OSA) with a higher risk of various cancers; however, causal relationships have not yet been definitively established.

**Methods:**

Our study evaluated the causal impact of OSA on the risk of developing 22 different types of cancer using univariable and multivariable Mendelian randomization (MR). OSA‐associated genetic instruments were obtained from the FinnGen study, which incorporates 38,998 OSA individuals and 336,659 non‐OSA individuals from European descent. Summary‐level data for 22 site‐specific cancers were estimated from large genetic consortia and UK Biobank. We used inverse‐variance weighting (IVW) as the primary analysis, along with several sensitivity analyses.

**Results:**

Univariable MR analyses indicated a causal relationship of genetic susceptibility to OSA on an increased risk of Barrett's esophagus (BE) and esophageal cancer (odds ratio [OR] = 1.32, 95% confidence interval [CI] = 1.07−1.62, *p* = 0.01), endometrial cancer (OR = 1.36, 95% CI = 1.16−1.60, *p* = 2.26E‐04), and its endometrioid subtype (OR = 1.28, 95% CI = 1.04−1.59, *p* = 0.02). Multivariable MR, accounting for possible confounders like drinking and smoking, confirmed the causal relationships of OSA on BE and esophageal cancer, and endometrial cancer.

**Conclusions:**

This study provided evidence regarding causal associations of OSA with higher risk of BE and esophageal cancer, and endometrial cancer.

## Graphical abstract

MR study of the effects of OSA on 22 site‐specific cancers.

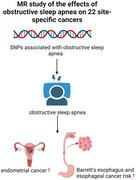

## 1 Introduction

Obstructive sleep apnea (OSA) is characterized by the recurrent obstruction of the upper airway while asleep, which causes intermittent hypoxia (IH), interrupted sleep, and disturbed physiological functioning (Jordan et al. 2014). The prevalence of OSA has increased with time and was reported in approximately 37% of men and in 50% of women globally (Franklin and Lindberg 2015). OSA is linked to an elevated risk of hypertension, metabolic dysfunction, and cardiovascular and cerebrovascular diseases (Giampá et al. 2023; Baltzis et al. 2016; Sánchez‐de‐la‐Torre et al. 2013).

Recent hypotheses suggest that the elevated mortality observed in OSA patients may be attributed not only to cardiometabolic effects but also to cancer. A multicenter retrospective cohort study, after adjusting for confounders, reported that severe OSA was linked to a 15% greater risk of cancer development compared to non‐OSA individuals (Kendzerska et al. 2021). Corroborating these findings, an analysis of a nationwide health insurance database identified significant associations between OSA and elevated incidence rates of pancreatic cancer, kidney cancer, and melanoma after adjusting for sex, age, and comorbidities (Gozal et al. 2016). Furthermore, a comprehensive meta‐analysis encompassing 5,276,451 participants revealed that OSA patients faced a 71% higher pooled risk of melanoma compared to their non‐OSA counterparts (Tan et al. 2021). Another meta‐analysis study highlighted a pronounced association between OSA and increased risks of kidney (HR = 1.75, 95% CI: 1.21–2.53) and bladder cancer (HR = 1.76, 95% CI: 1.05–2.96), though no significant correlation was found with prostate cancer (Yeo et al. 2024). Evidence from clinical and animal model studies further supports the association of OSA with cancer progression and metastasis, potentially mediated by IH, which promotes tumor angiogenesis, growth, and cell proliferation (Almendros et al. 2012, 2014; Toffoli and Michiels 2008; Harris 2002; Carmeliet et al. 1998). However, previous observational studies are limited by small sample sizes, reverse causation bias, and confounding factors such as obesity, smoking, and drinking, which are prevalent risks for both OSA and cancer.

Mendelian randomization (MR) provides a method to mitigate these biases by using genetic variants to assess causal associations between exposures and outcomes (Emdin et al. 2017). By leveraging the random assortment of genetic variants during meiosis, MR ensures that instrumental variables (IVs) are independent of potential confounders, such as age, sex, lifestyle factors, and environmental influences. This approach mimics the design of a randomized controlled trial, effectively reducing bias and providing a more robust framework for causal inference (Sang et al. 2022; Chen et al. 2021; Park et al. 2020). While a prior MR study established a causal relationship of OSA on increased breast cancer risk, it did not explore the impact of OSA on other cancer sites (Gao et al. 2020). We systematically evaluated the causal relationship between OSA and the risk of 22 site‐specific cancers in this study.

## 2 Materials and Methods

### 2.1 Study Design

**FIGURE 1:**
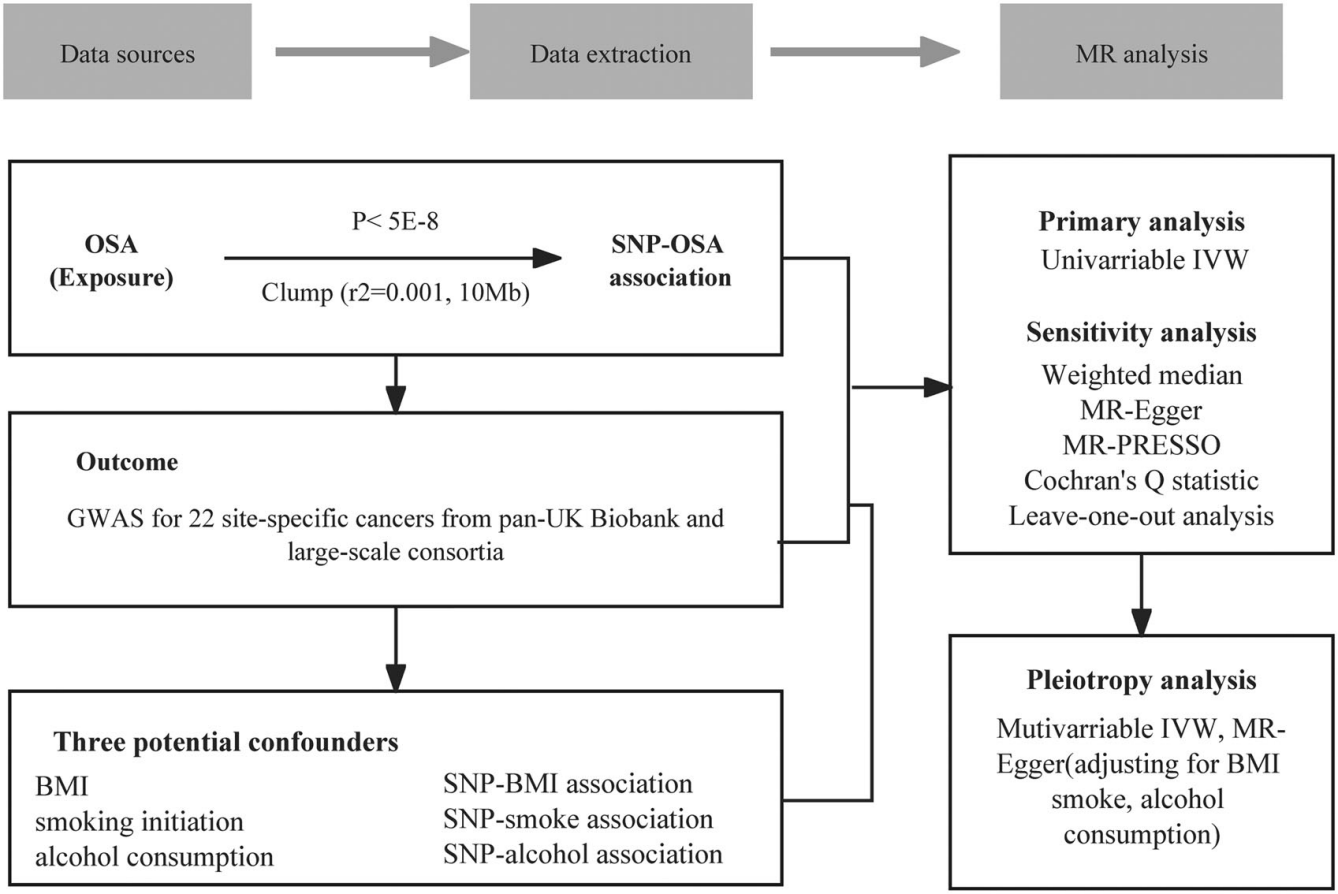
The flow chart depicts the study design and the MR analysis procedure.

This study employs a two‐sample MR to investigate the causal link between OSA and cancer risk. The validity of MR hinges on three fundamental principles: (1) the genetic variants used as IVs must exhibit a strong and reliable association with OSA; (2) the IVs must be independent of potential confounders; and (3) the IVs must affect the outcome (cancer risk) exclusively through OSA, without alternative pathways. The flow chart of this study is shown in Figure 1.

### 2.2 Data Source

The summary statistics for OSA was derived from the FinnGen consortium's R9 release (https://www.finngen.fi/en), which included 38,998 OSA patients and 336,659 controls of European descent. The diagnosis of OSA was determined using the International Classification of Diseases, Ninth Revision (ICD‐9: 3472A) and Tenth Revision (ICD‐10: G47.3) codes, obtained from the Finnish National Hospital Discharge Registry and the Causes of Death Registry. OSA diagnosis was based on subjective symptoms, clinical examination, and sleep studies, with a threshold of either an apnea‐hypopnea index (AHI) ≥ 5 events/h or a respiratory event index (REI) ≥ 5 events/h (Strausz et al. 2021).

Summary‐level data for cancer outcomes were available from pan‐UK Biobank (Sudlow et al. 2015) and large‐scale consortia (Zhang et al. 2020; O'Mara et al. 2018; Yang et al. 2022). The detailed information on cancers was exhibited in Table S1. Additionally, we utilized the multivariable MR (MVMR) to evaluate the direct influence of OSA on cancer risk, taking into consideration confounders such as smoking, drinking, and body mass index (BMI). Summary statistics on smoking initiation (311,629 cases vs. 321,173 controls) and alcohol intake (335,394 samples) were obtained from the GWAS and the Alcohol and Nicotine Use Sequencing Consortium (Liu et al. 2019). BMI statistics were derived from the GWAS meta‐analysis of approximately 700,000 European participants (Yengo et al. 2018). As all summary statistics from GWAS were accessible to the public, ethical approval was not required for this research.

### 2.3 Selection of IVs

In order to guarantee the genuineness and precision of the findings regarding the connection between OSA and cancer risk, the subsequent measures of quality control were employed in order to choose the most suitable IVs. Initially, single nucleotide polymorphisms (SNPs) below the threshold of statistical significance across the entire genome (5 × 10−8) were regarded as IVs. Next, we conducted the clumping procedure to evaluate linkage disequilibrium (LD) among the selected SNPs, employing an *R* ^2^ threshold of 0.001 and a clumping distance of 10 Mb. For SNPs present in the exposure data but not in the outcome data, the clustering method using proxies (LD *R* ^2^ value ≥ 0.8) was applied. Subsequently, we coordinated the exposure and outcome datasets to exclude SNPs with palindromic effects and allelic inconsistencies. Lastly, the *F* statistics were calculated as follows: *F* = [(N − *k* − 1)/*k*]/[*R*2/(1 − *R*2)] (Burgess and Thompson 2017), and weak IVs with *F* < 10 are eliminated. *R*2 represented the proportion of variance in the exposure explained by the IVs. Specific information on the IVs related to OSA is presented in Table S2.

### 2.4 Statistical Analysis

The primary evaluation method for assessing the correlation between genetically predicted OSA and cancer outcomes is the inverse‐variance weighted (IVW) analysis, which offers the most dependable causal estimates but was susceptible to pleiotropic studies (Burgess et al. 2013). The weighted median method provided accurate estimates of causal effects when over 50% of the weight comes from valid SNPs (Bowden et al. 2016). We performed comprehensive sensitivity analyses to verify the findings of the MR analysis. Initially, the intercept of the MR‐Egger regression was used to assess horizontal pleiotropy (Bowden et al. 2015). Subsequently, the MR pleiotropy residual sum and outlier (MR‐PRESSO) analysis was used to assess multipotency, which involves identifying and removing outliers to address horizontal pleiotropy, and to evaluate if there are significant differences in causal effects before and after outliers are removed (Verbanck et al. 2018). Moreover, to evaluate the heterogeneity among SNPs associated with OSA, Cochran's *Q* test was employed. Finally, the “leave‐one‐out” analysis was utilized to determine if the impact of eliminating particular SNPs influenced the effect estimates (Figure S1).

The MVMR method adjusted for potential confounders has a distinct advantage in favor of specifying the direct effect of OSA on cancers. Given that BMI, smoking, and drinking are strongly correlated with OSA and are associated with an increased risk of cancer, we employed MVMR analysis to assess the direct impact of OSA on cancer risk, independent of these confounding factors. Detailed information about the IVs associated with these confounders (smoking, drinking, and BMI) are provided in Tables S3–S5. The MVMR‐IVW method (Burgess and Thompson 2015) and the MVMR‐MR Egger method (Rees et al. 2017) were utilized for conducting the MVMR analyses. Additionally, we evaluated horizontal pleiotropy by performing the MR‐Egger intercept test.

The statistical analysis and visualization of results were performed using the R statistical programme (version 4.1.2). A *p*‐value < 0.05 was deemed statistically significant for the outcomes of MR and sensitivity analyses. After adjusting for multiple comparisons, a *p*‐value < 0.025 was deemed significant.

## 3 Results

### 3.1 Univariable MR Results

**FIGURE 2:**
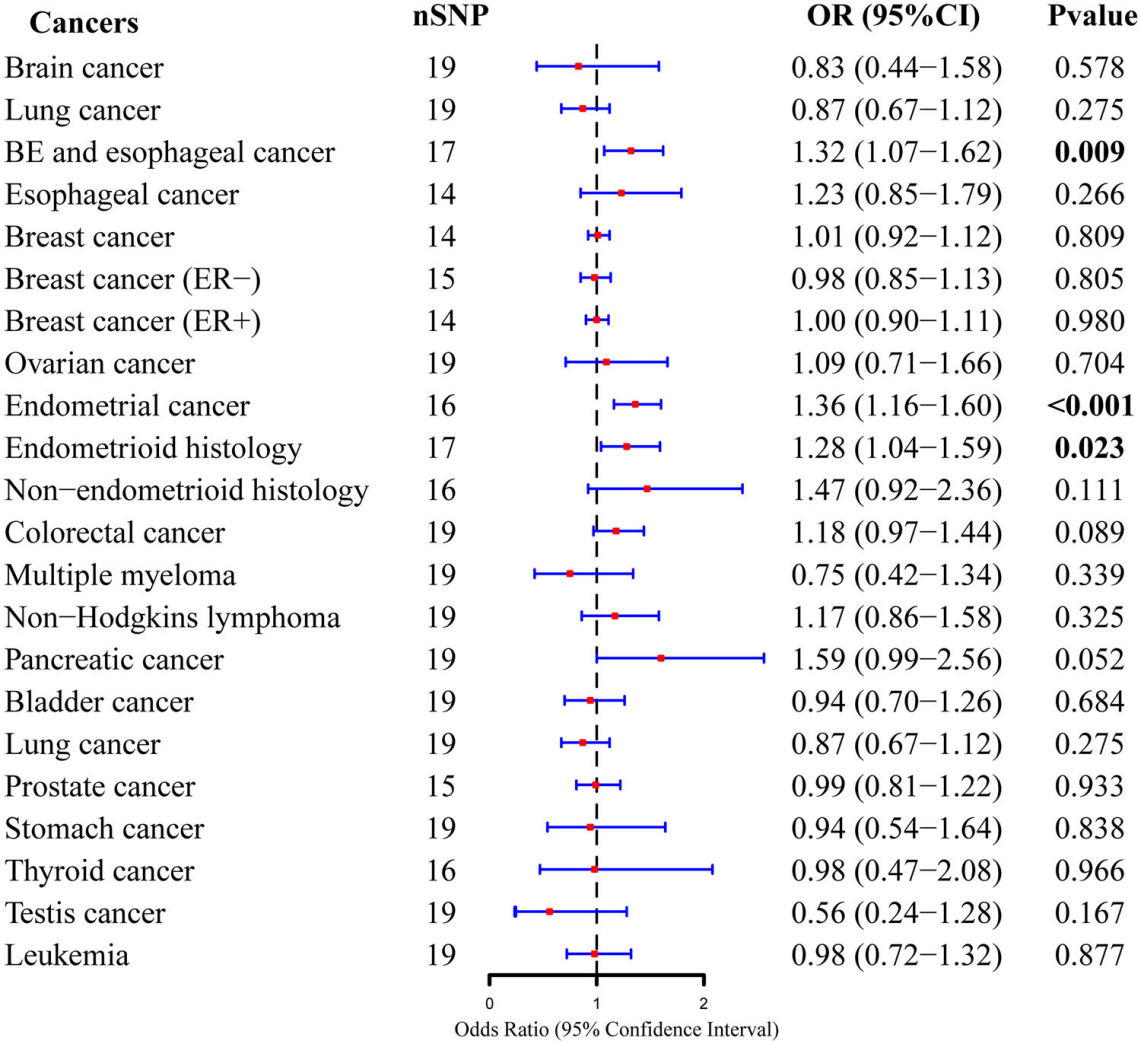
Forest plot of the causal association between OSA and 22 site‐specific cancers as predicted by IVW.

We identified 20 independent genetic instruments for OSA which together explained close to 4.6% of the phenotypic variation in OSA (Table S2). The effects of OSA on cancer risk as predicted by IVW are shown in Figure 2. The UVMR method showed that OSA was significantly linked with a higher risk of BE and esophageal cancer (OR = 1.32, 95% CI = 1.07–1.62, *p* = 0.01), endometrial cancer (OR = 1.36, 95% CI = 1.16–1.60, *p* = 2.26E‐04), and endometrioid endometrial cancer (OR = 1.28, 95% CI = 1.04–1.59, *p* = 0.02). Using the weighted median method, evidence showed that the estimates of the causal relationship between OSA and endometrial cancer (OR = 1.35, 95% CI = 1.08–1.70, *p* = 0.01), endometrioid endometrial cancer (OR = 1.45, 95% CI = 1.10–1.92, *p* = 0.01), as well as BE and esophageal cancer (OR = 1.34, 95% CI = 1.01–1.80, *p* = 0.04) are consistent in direction with IVW (Table S6). The MR‐Egger intercept test showed no notable deviation from zero, suggesting no horizontal pleiotropy. After removing outliers, the MR‐PRESSO global test suggested the absence of substantial horizontal pleiotropy for IVs, indicating the IVW results are reliable (Table S7). Cochran's *Q* test demonstrated no substantial heterogeneity among IVs. Furthermore, the leave‐one‐out analysis demonstrated that no single SNP affected the MR results (Figure S1).

### 3.2 MVMR Results

**FIGURE 3:**
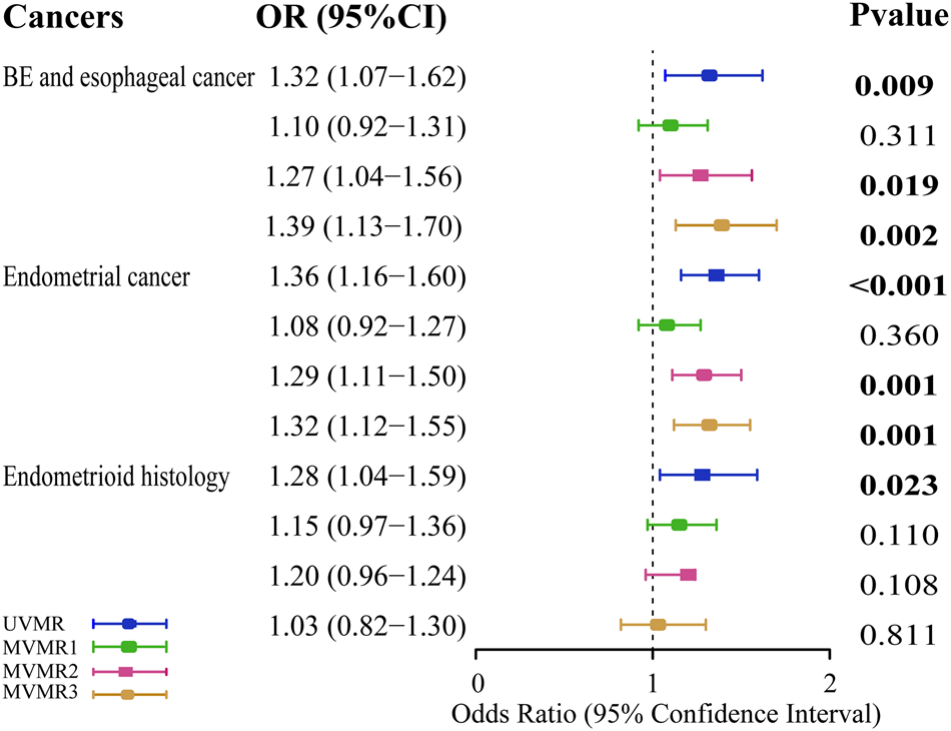
Forest plot of the causal association between OSA and cancers. MVMR1: MVMR results adjusted for BMI; MVMR2: MVMR results adjusted for smoking; MVMR3: MVMR results adjusted for alcohol consumption.

As shown in Figure 3, further MVMR analysis indicated that OSA still had a positive direct effect on BE and esophageal cancer (controlling for smoking: OR_IVW_ = 1.27, 95% CI = 1.04–1.56, *p* = 0.019; controlling for alcohol consumption: OR_IVW_ = 1.39, 95% CI = 1.13–1.70, *p* = 0.002) and endometrial cancer (controlling for smoking: OR_IVW_ = 1.29, 95% CI = 1.11–1.50, *p* = 0.001; controlling for alcohol consumption: OR_IVW_ = 1.32, 95% CI = 1.12–1.55, *p* = 0.001). However, after accounting for BMI, no significant direct effect was detected for OSA on the risk of BE and esophageal cancer (OR_IVW_ = 1.10, 95% CI = 0.92–1.31, *p* = 0.311), endometrial cancer (OR_IVW_ = 1.08, 95% CI = 0.92–1.27, *p* = 0.360) and endometrioid endometrial cancer (OR_IVW_ = 1.15, 95% CI = 0.97–1.36, *p* = 0.110). Furthermore, the intercept term derived from MR‐Egger failed to identify horizontal pleiotropy (Table S8).

## 4 Discussion

In our MR investigation, we systematically examined the causative relationships between OSA and 22 specific types of cancer. We found evidence that OSA was linked to an increased risk of endometrial cancer, endometrioid endometrial cancer, and BE and esophageal cancer.

Several research have shown inconsistent conclusions about the link between OSA and cancer risk. A recent meta‐analysis of 34,848 individuals with OSA and 77,380 control subjects found that OSA significantly increases the risk of developing cancer (RR = 1.53, 95% CI = 1.31–1.79, *p* < 0.001). After adjusting for conventional cancer risk variables, the correlation weakened but remained statistically significant (RR = 1.40, 95% CI = 1.01–1.95, *p* < 0.04) (Palamaner Subash Shantha et al. 2015). In a retrospective cohort study from China, researchers discovered that individuals with OSA exhibited a significantly higher risk of cancer development compared to those without OSA. Furthermore, the severity of OSA was found to have a significant positive correlation with cancer incidence (Xiong et al. 2022). Nevertheless, Christensen et al. conducted a prospective study with 8783 participants and were unable to replicate these findings. They observed no association between OSA and cancer incidence at either the individual or overall level (Christensen et al. 2013). Epidemiological studies of relationships of OSA and the incidence of specific cancer sites are limited and inconsistent (Choi et al. 2020; Lee et al. 2022; Carreres et al. 2022; Huang et al. 2021; Chen et al. 2022). The discrepancy in results could be explained by high heterogeneity of some retrospective studies, residual confounding factors, reverse causality, or reporting bias.

The association between OSA and specific tumor types is equally contentious. For instance, research on colorectal cancer has produced mixed results. While some studies report a 1.8‐fold increased risk of colorectal cancer in OSA patients compared to controls (Kendzerska et al. 2021; Chen et al. 2020), others paradoxically suggest a potential protective effect of OSA against colorectal cancer development (Sillah et al. 2018). Similarly, the relationship between OSA and kidney cancer remains highly debated. Although several studies have identified a significantly elevated risk of kidney cancer in OSA patients (Gozal et al. 2016; Yeo et al. 2024), Kendzerska et al. (2021) found no meaningful association between the two conditions. Additionally, the potential links between OSA and other malignancies, including prostate, breast, lung, and pancreatic cancers, remain unresolved, with the existing literature presenting contradictory evidence (Kendzerska et al. 2021; Gozal et al. 2016; Choi et al. 2019; Yan et al. 2024; Cheong et al. 2022; Sircu et al. 2023).

The main problem in previous studies is that the methodology is not ideal, because most of these studies were retrospective studies and originally designed for other purposes. Another drawback is that some cohort studies used less reliable sleep‐testing devices to define OSA. Furthermore, most research have investigated the link between OSA and overall cancer risk, ignoring the link with site‐specific cancers. In the event that OSA was to demonstrate a connection with the occurrence or advancement of cancer, it would remain uncertain whether this correlation is restricted to particular cancer locations or categories of malignant cells, or if it applies universally to all forms of cancer.

Our findings for OSA and BE and esophageal cancer partly support the results from a case–control study of 7482 participants, which observed that individuals with OSA had approximately 80% increased risk for BE to controls without OSA (OR = 1.8; 95%CI = 1.1–3.2; *p* = 0.03). The association between OSA and BE remained unchanged in a multivariable model that included smoking history, BMI and GERD (Leggett et al. 2014). In the current study, we also identified a significant causal relationship between genetic susceptibility to OSA and an increased risk of endometrial cancer. However, it is crucial to highlight that after adjusting for BMI, the associations between OSA and both endometrial cancer and esophageal cancer (including BE) were no longer statistically significant, suggesting that obesity may confound these relationships. Further investigation is required to elucidate the mechanisms behind the association between OSA and endometrial cancer. While a previous MR study conducted on an Asian population discovered a significant and positive correlation between OSA and breast cancer risk, the present study offers very minimal evidence to establish the existence of a causal association between the two. This could perhaps be attributed to racial disparities (Gao et al. 2020).

The precise mechanisms by which OSA contributes to cancer development remain incompletely understood. However, several potential pathways have been proposed, with IH emerging as a key factor (Abrams 2007). IH plays a critical role in shaping the tumor microenvironment by promoting the recruitment of macrophages and altering their polarity. Specifically, IH shifts macrophages toward a tumor‐promoting phenotype (M2 polarization) rather than a tumor‐suppressing one (M1 polarization) (Ruffell et al. 2012). Preclinical studies have demonstrated that tumor‐associated macrophages (TAMs) from IH‐exposed mice exhibit a more pronounced ability to enhance cancer cell proliferation, migration, and extravasation compared to those from normoxic or normal sleep conditions. At the cellular level, hypoxia induces metabolic reprogramming, leading to the upregulation of hypoxia‐inducible factors (HIFs) and other regulators that promote inflammation, cellular dysfunction, and impaired apoptosis (Harris 2002; Semenza 2012). Additionally, hypoxia‐generated oxidative stress causes DNA oxidation damage, which inhibits DNA repair mechanisms and facilitates the accumulation of mutations (Hamanaka and Chandel 2010). These genetic alterations, including mutations in genes such as TP53, KRAS, PIK3CA, BRCA1, and ATM, contribute to the malignant transformation of cells (Pouysségur et al. 2006). OSA further exacerbates these effects by sustaining elevated hypoxia‐inducible factor‐1 (HIF‐1) activity in both tumors and their surroundings. HIF‐1 overexpression not only promotes angiogenesis through the upregulation of vascular endothelial growth factor (VEGF), facilitating tumor growth and metastasis, but also modulates gene expression by activating key transcription factors. Notably, activator protein‐1 (AP‐1) and nuclear factor‐κB (NF‐κB) are central to these processes. AP‐1, which includes viral oncoproteins, drives cellular proliferation and survival, while NF‐κB stimulates anti‐apoptotic factors, proliferation molecules, and pro‐angiogenic factors, further supporting cancer progression (Toffoli and Michiels 2008; Reuter et al. 2010).

This work employs a variety of complementary MR methods to examine the causal link between genetically predicted OSA and 22 particular types of cancer. The MR analysis has the advantage of being less prone to bias and reverse causality compared to observational investigations. Furthermore, the results remain consistent in various sensitivity analyses and MVMR analyses, indicating the reliability and persuasiveness of the results. Several limitations of our study should be acknowledged. First, the sample data used for our study originated exclusively from individuals of European descent. Although this approach helps minimize the influence of demographic stratification, it is crucial to emphasize that our results may not apply to other ethnic populations. Second, although MVMR accounted for BMI, smoking, and alcohol consumption, residual confounding from other OSA‐related comorbidities (e.g., type 2 diabetes and hypertension) remains possible. Notably, while BMI adjustment partially addresses obesity, it may not fully reflect its downstream metabolic consequences, including insulin resistance and dysregulated adipokine secretion, which are independently linked to cancer risk (Calle and Kaaks 2004). Third, as OSA diagnoses were primarily based on clinical records, detailed information on sleep study protocols and scoring criteria was unavailable. Fourth, we were unable to stratify our analysis using the AHI to categorize OSA severity into mild, moderate, or severe. As highlighted in the meta‐analysis (Tan et al. 2022; Cao et al. 2022), severe OSA is a critical determinant of cancer risk, and future studies should incorporate measures of OSA severity, including AHI, oxygen desaturation index (ODI), and nocturnal hypoxemia, to provide a more comprehensive understanding of the relationship between OSA and cancer risk. Finally, despite employing rigorous methods to detect and account for pleiotropic variants, we cannot entirely eliminate the potential influence of pleiotropy, given the complex and often ambiguous biological roles of many genetic variants.

## 5 Conclusions

Overall, our findings provide novel evidence that a genetic predisposition to OSA is linked to an elevated risk of endometrial cancer, BE and esophageal cancer.

## Author Contributions

**Xiaoyu He**: writing–original draft, formal analysis, data curation. **Xiaoting Ye**: writing–review and editing. **Kaiqian Yang**: writing–review and editing, visualization. **Zhenjiang Li**: funding acquisition.

## Conflicts of Interest

The authors declare no conflicts of interest.

### Peer Review

The peer review history for this article is available at https://publons.com/publon/10.1002/brb3.70462

## Supporting information



Supporting Information.

Supporting Information

## Data Availability

The authors affirm that all data supporting the conclusions are either readily accessible on websites without any limitations or can be obtained from consortia upon application.
