# Supplementary material for: Causal Associations of Obstructive Sleep Apnea With Cancer Risk: A Mendelian Randomization Study
Source: Brain Behav. 2025 May 5;15(5):e70462. doi: 10.1002/brb3.70462 (PMC12050956; doi:10.1002/brb3.70462)
Supplement: Supplementary file 1 — Supporting Information. [file BRB3-15-e70462-s002.pdf]

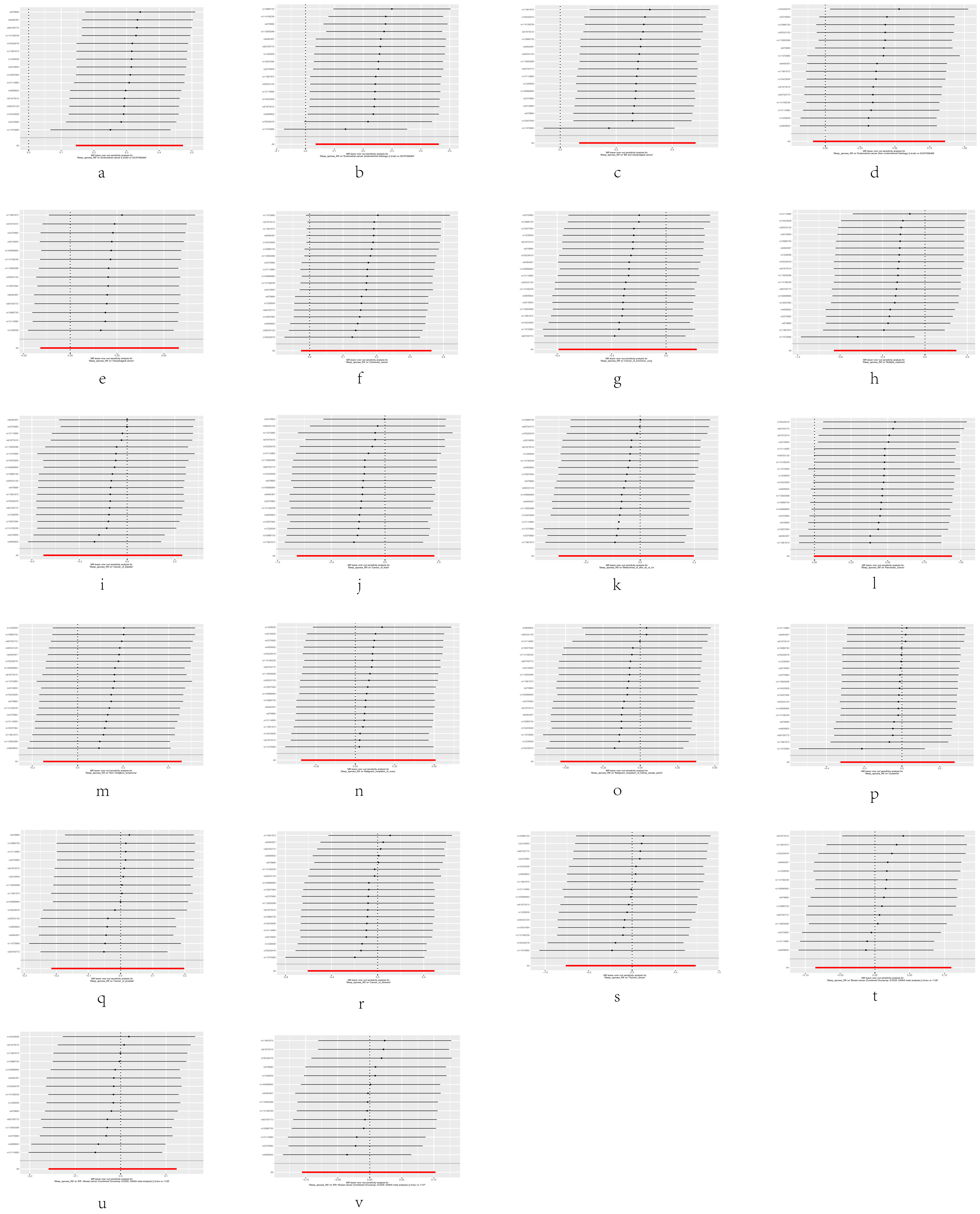

Supplementary Figure 1. MR leave-one-out sensitivity analyses for OSA on cancers

(a) MR leave-one-out sensitivity analysis for OSA on endometrial cancer.

(b) MR leave-one-out sensitivity analysis for OSA on endometrial cancer (endometrioid histology).

(c) MR leave-one-out sensitivity analysis for OSA on BE and esophagus cancer.

(d) MR leave-one-out sensitivity analysis for OSA on endometrial cancer (non-endometrioid histology).

(e) MR leave-one-out sensitivity analysis for OSA on esophagus cancer.

(f) MR leave-one-out sensitivity analysis for OSA on colorectal cancer.

(g) MR leave-one-out sensitivity analysis for OSA on lung cancer.

(h) MR leave-one-out sensitivity analysis for OSA on MM.

(i) MR leave-one-out sensitivity analysis for OSA on bladder cancer.

(j) MR leave-one-out sensitivity analysis for OSA on brain cancer.

(k) MR leave-one-out sensitivity analysis for OSA on melanomas.

(l) MR leave-one-out sensitivity analysis for OSA on pancreatic cancer.

(m) MR leave-one-out sensitivity analysis for OSA on NHL.

(n) MR leave-one-out sensitivity analysis for OSA on ovarian cancer.

(o) MR leave-one-out sensitivity analysis for OSA on kidney cancer.

(p) MR leave-one-out sensitivity analysis for OSA on leukemia .

(q) MR leave-one-out sensitivity analysis for OSA on prostate cancer.

(r)MR leave-one-out sensitivity analysis for OSA on stomach cancer.

(s)MR leave-one-out sensitivity analysis for OSA on thyroid cancer.

(t)MR leave-one-out sensitivity analysis for OSA on breast cancer.

(u)MR leave-one-out sensitivity analysis for OSA on breast cancer (ER-) .

(v)MR leave-one-out sensitivity analysis for OSA on breast cancer (ER+).

Abbreviations: OSA,obstructive sleep apnea;BE,barrett's esophagus;MM,multiple myeloma;NHL,non hodgkins lymphoma;ER+,estrogen receptor-positive;ER-,estrogen receptor-negative.
